# Supplementary material for: The mechanisms to dispose of misfolded proteins in the endoplasmic reticulum of adipocytes
Source: Nat Commun. 2023 May 30;14:3132. doi: 10.1038/s41467-023-38690-4 (PMC10229581; doi:10.1038/s41467-023-38690-4)
Supplement: Supplementary file 1 — Supplementary Information [file 41467_2023_38690_MOESM1_ESM.pdf]

Supplementary Information

The mechanisms to dispose of misfolded proteins in the endoplasmic reticulum of adipocytes

Shuangcheng Alivia Wu[1], Chenchen Shen[2], Xiaoqiong Wei[1], Xiawei Zhang[1], Siwen Wang[1], Xinxin Chen[1], Mauricio Torres[1], You Lu[1], Liangguang Leo Lin[1], Huilun Helen Wang[1], Allen H. Hunter[3], Deyu Fang[4], Shengyi Sun[5], Magdalena I. Ivanova[6,7], Yi Lin[2 *] and Ling Qi[1,8 *]

This PDF file includes:

Supplementary Figures 1-10

Supplementary Tables 1-3

Supplementary Figures and Legends

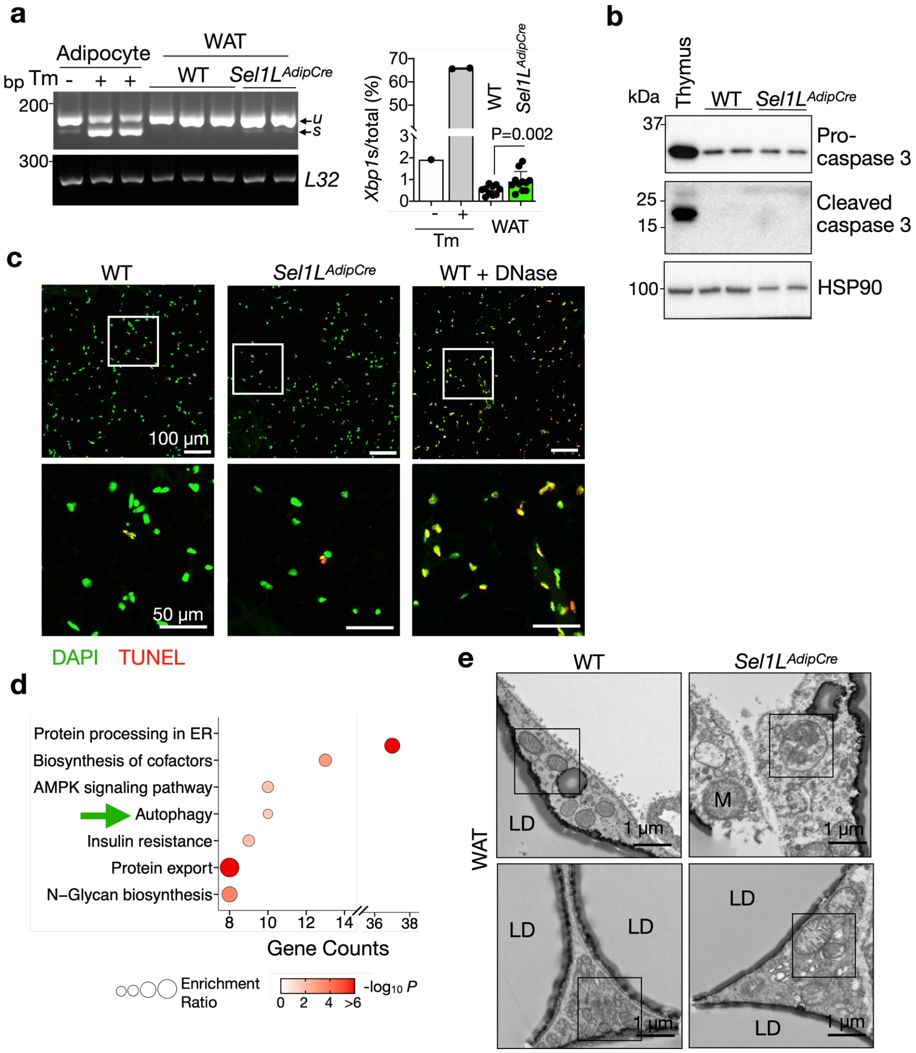

Supplementary Figure 1. Cellular adaption and activation of autophagy in *Sel1L*-deficient adipocytes.

a RT-PCR analysis of *Xbp1* mRNA splicing in gonadal WAT of 12-week-old mice with quantitation shown on the right (n=10 for WT and 11 for *Sel1L*^{AdipCre}). WT adipocytes treated with tunicamycin (Tm), positive controls. **b** Immunoblot analysis of (cleaved) caspase 3 in gonadal WAT of 12-week-old mice (n=4 mice per genotype). Mouse thymus, a positive control. **c** Representative confocal images of TUNEL staining in gonadal WAT of 12-week-old mice mice (n=2 mice per genotype). WT WAT section treated with DNase, a positive control. **d** Top upregulated pathways in WAT of *Sel1L*^{AdipCre} versus WT mice using the KEGG pathway analysis of Affymetrix microarray data (n=4 mice per genotype). **e** TEM images of gonadal WAT of WT and *Sel1L*^{AdipCre} mice at the age of 12 weeks (n=3 mice per genotype) with boxed area shown in Fig 1b. LD: lipid droplet; M: mitochondria. Histogram was plotted as mean with SD; each data points were derived from biologically independent mice. P values were derived by two-sided Student's *t*-test (a) and two-sided Fisher's Exact Test (d).

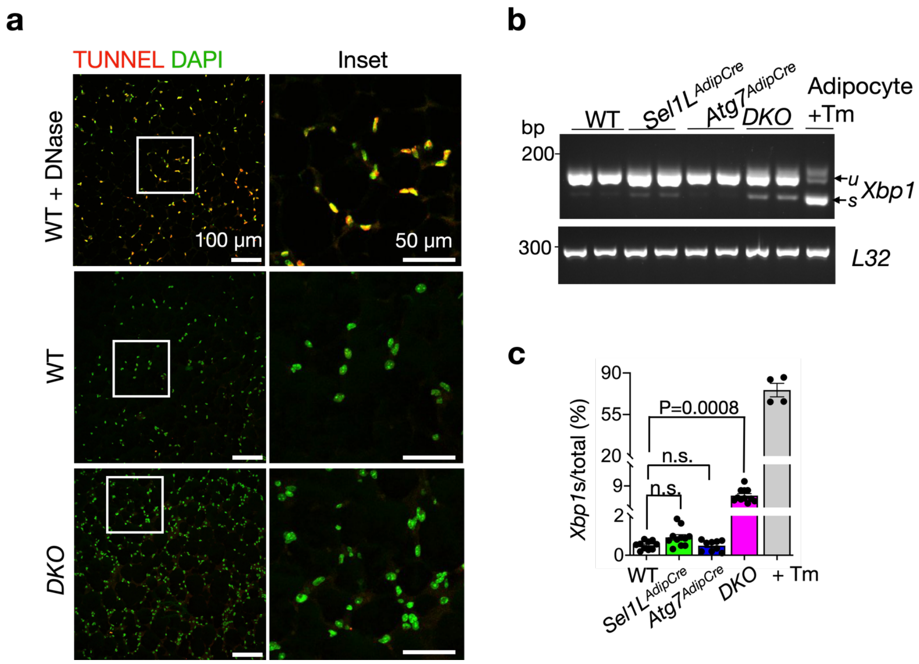

Supplementary Figure 2. Adipocytes are able to adapt to the lack of both SEL1L-HRD1 ERAD and autophagy.
a Representative confocal images of TUNEL staining in gonadal WAT of WT and *DKO* mice at the age of 12 weeks (n=2 mice per genotype). **b** RT-PCR analysis of *Xbp1* mRNA splicing in gonadal WAT of 12-week-old mice with quantitation shown in **c** (n=10 for WT, 9 for $Atg7^{AdipCre}$, 11 for $Sel1L^{AdipCre}$ and *DKO*). Histogram was plotted as mean with SD; each data points were derived from biologically independent mice. P values were derived by by One-way ANOVA followed by Tukey's test; n.s., not significant.

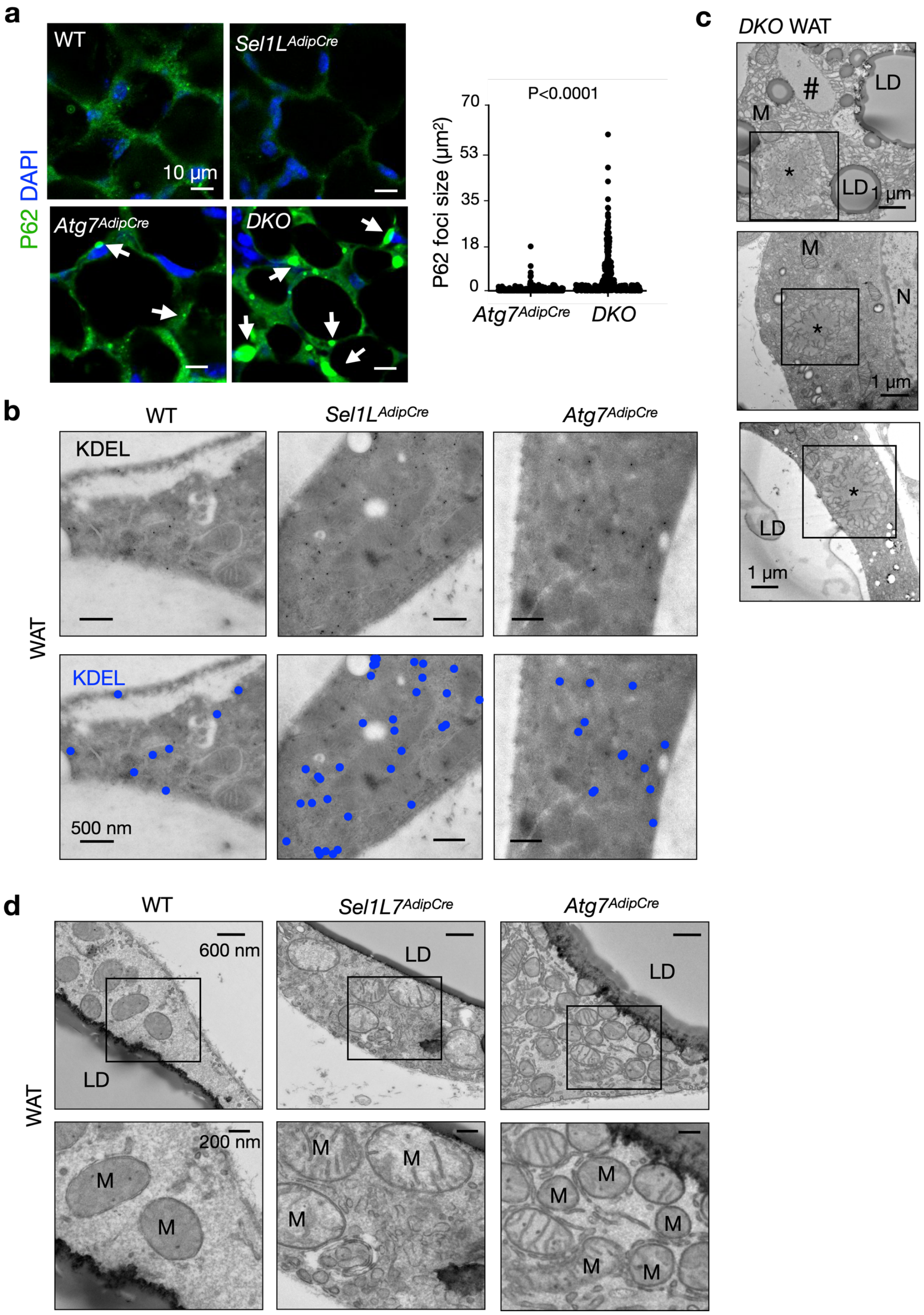

Supplementary Figure 3. Coalescence of ER fragments (CERFs) in *DKO* adipocytes.
a Representative immunofluorescent images of P62 in gonadal WAT with quantitation of P62 inclusion size shown on the right (n=3 mice per genotype). Arrows, P62 inclusions. P value was derived by two sided Student's *t*-test. **b** Representative EM images following immunogold labeling of the ER marker KDEL in gonadal WAT (n=2-3 mice per genotype). Color-coded images show gold particles. **c** Representative TEM image of *DKO* gonadal WAT (n=3 mice). Boxed images are shown in Fig. 2d. **d** Representative TEM images of gonadal WAT (n=2-4 mice per genotype. #, P62 inclusion; *, CERF; LD, lipid droplet; M, mitochondria; N, nucleus.

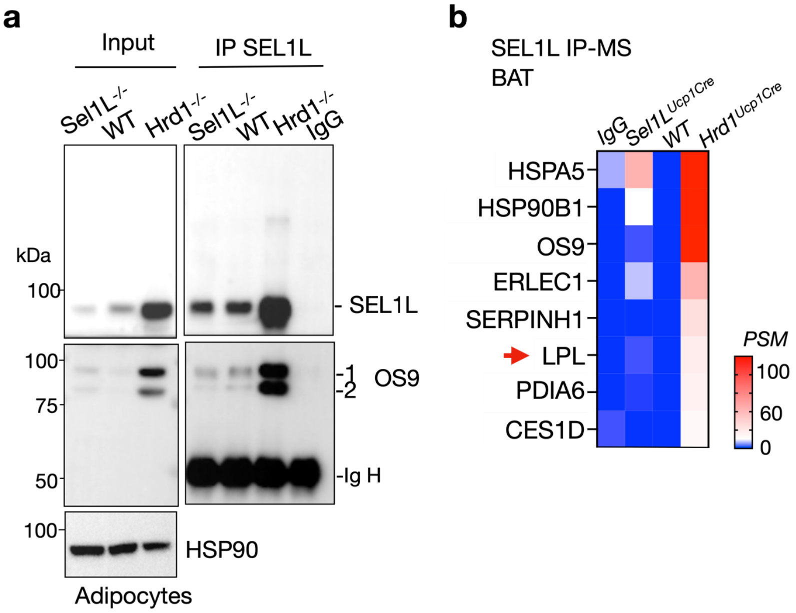

Supplementary Figure 4. IP-MS identification of LPL as a candidate of endogenous SEL1L-HRD1 ERAD substrates.

a Validation of SEL1L antibody in immunoprecipitation (IP) in WT and CRISPR CAS9-generated Hrd1 knockout (*Hrd1*$^{-/-}$) adipocytes after differentiation (n=2 independent repeats). *Sel1L*$^{-/-}$ mature adipocytes and IgG were used as negative controls. **b** Top protein hits identified by LC-MS analysis of SEL1L IP in BAT. PSM, peptide spectrum matches. The criteria and full list of hits were provided in Supplementary table 1. The SEL1L IP-MS was performed once using BAT from WT, *Hrd1*^{Ucp1Cre} and *Sel1L*^{Ucp1Cre} mice.

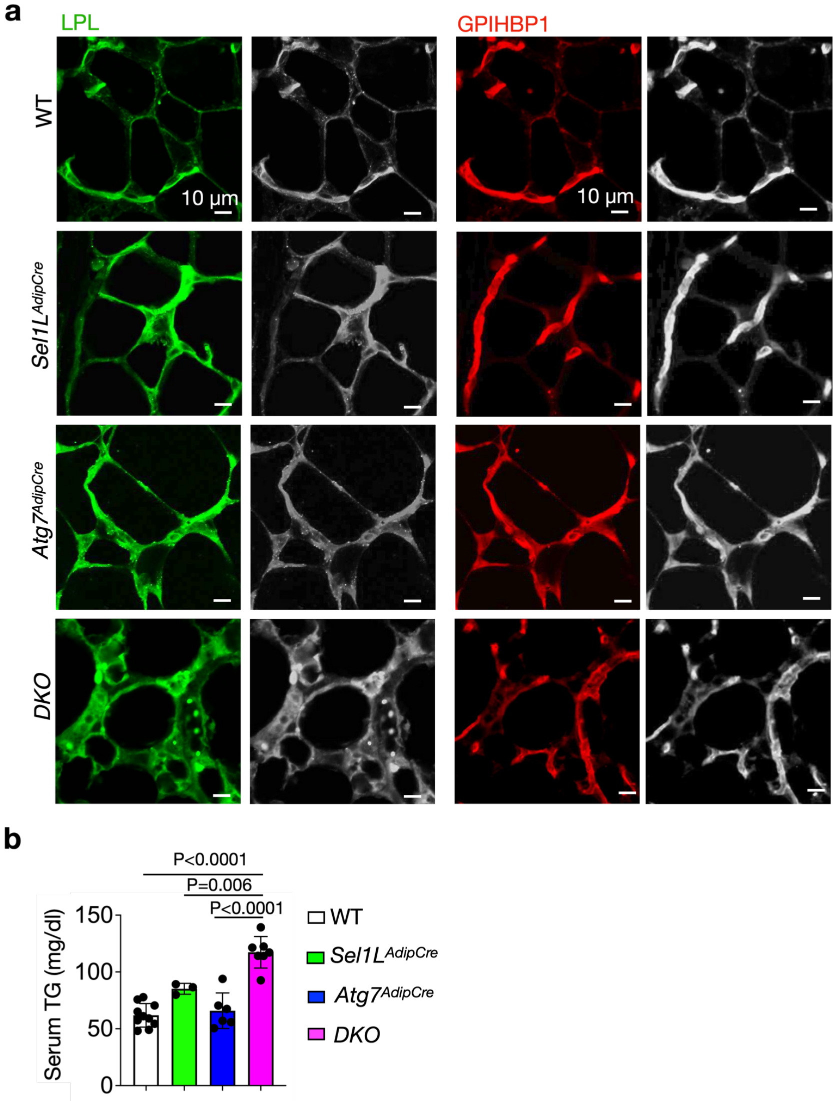

Supplementary Figure 5. LPL failed to reach capillary in the absence of ERAD.
a Single-channel and grayscale images of immunofluorescent co-labeling of LPL and the endothelial marker GPIHBP1 in gonadal WAT shown in Fig. 4d. **b** Serum triglycerides (TG) levels of mice after oral gavage of olive oil for 2 hr (n=10 for WT, 3 for *Sel1L^{AdipCre}*, 6 for *Atg7^{AdipCre}* and 7 for *DKO*). Histogram was plotted as mean with SD; each data points were derived from biologically independent mice. P value was derived by one-way ANOVA followed by Tukey's test; n.s. not significant.

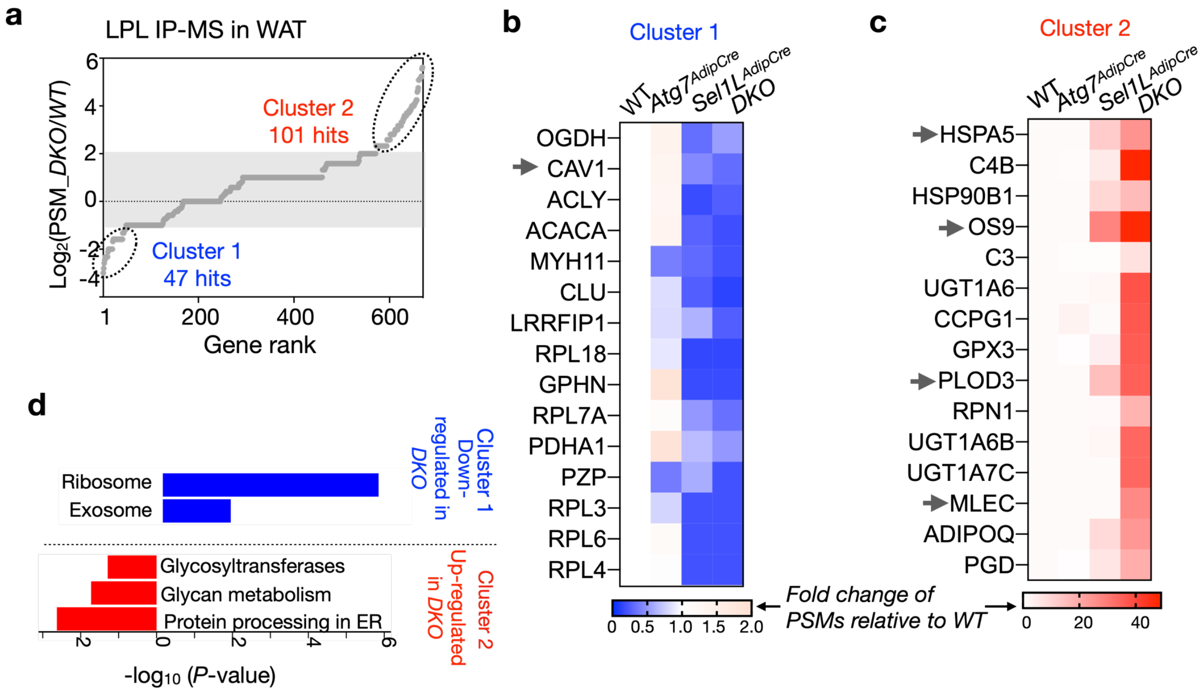

Supplementary Figure 6. LPL is associated with multiple ER chaperones in the CERFs.
a-d LC-MS analysis of LPL-IP in gonadal WAT in two experiments (one with pooled WAT from n=5 WT mice, 6 *Sel1L*^{AdipCre} mice and 7 *DKO* mice, and the other with n=4 WT mice and 3 *Atg7*^{AdipCre} mice): **a** Log2 fold change of PSMs (Peptide Spectrum Matches) from *DKO* mice compared to those of WT mice, highlighting two different clusters (1 and 2) identified in *DKO* vs. WT samples. **b-c** Heatmap of top hits in clusters 1 (**b**) and 2 (**c**), showing the fold change of PSMs of each sample to those of WT samples in each experiment. A complete list of hits and selection criteria shown in Supplementary Tables 2-3. Arrows point to those validated in Fig. 5. **d** Pathway analysis based on protein hits in clusters 1 and 2. P values are calculated by two-tailed rate ratio tests and adjusted for multiple testing by False Discovery Rate (FDR).

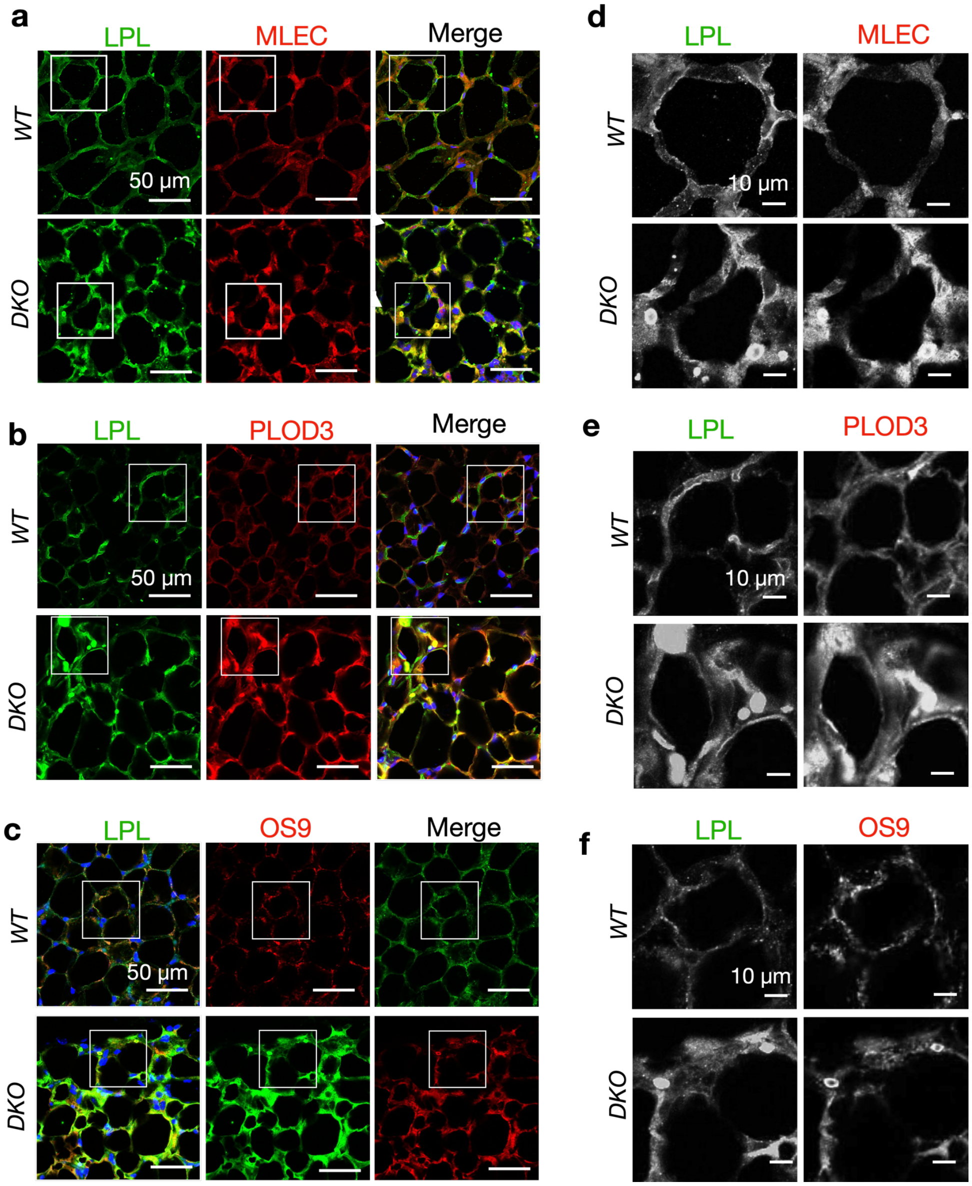

Supplementary Figure 7. LPL is associated with multiple ER chaperones in the CERFs.
a-c Immunofluorescent images of co-labeling of LPL and cluster 2 hits with merged boxed images shown in Fig. 5c-e. **d-f** Grayscale of immunofluorescent images shown in Fig. 5c-e. (n=3 mice per genotype for PLOD3, n=2 mice per genotype for MLEC and n=2 mice per genotype for OS9).

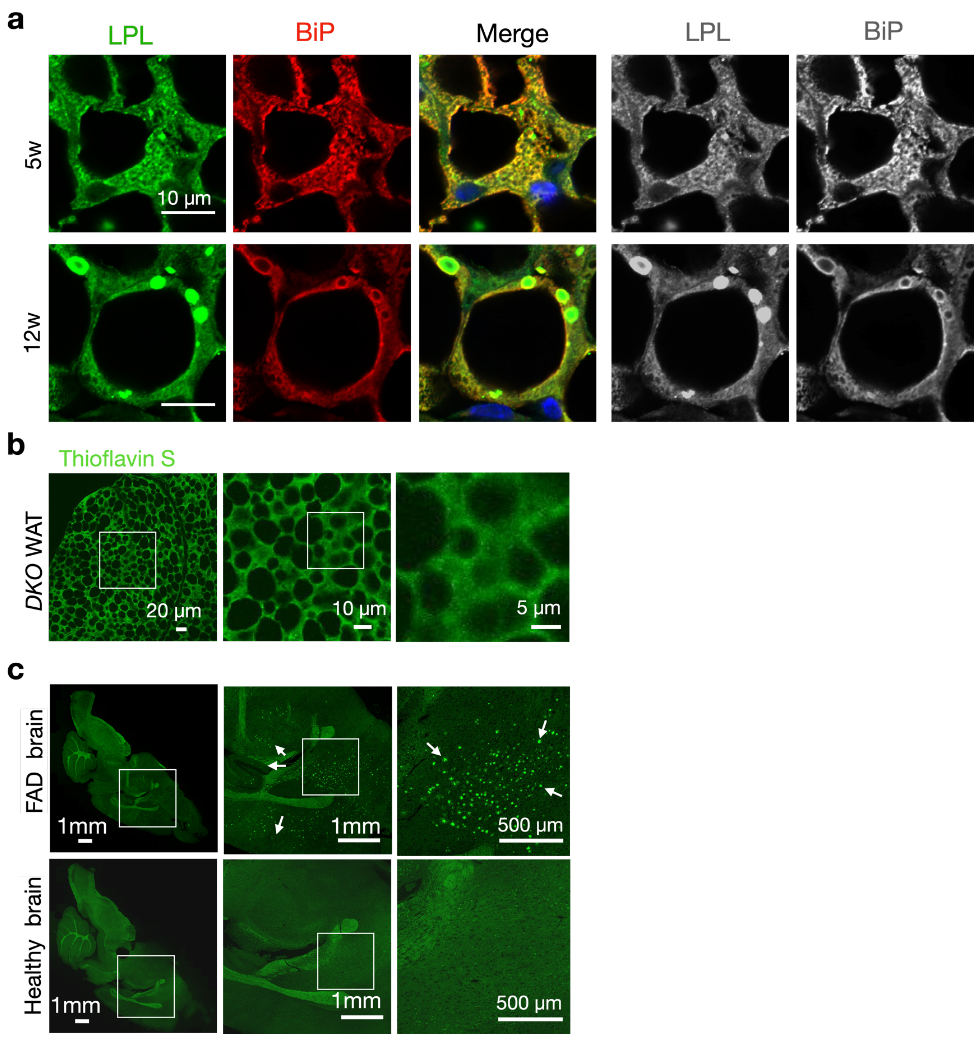

Supplementary Figure 8. CERFs are not amyloidogenic.
a Single-channel and grayscale of immunofluorescence images shown in Fig. 6e (n=3 mice per group).
b-c Representative immunofluorescent images of thioflavin S (ThS) staining in (b) gonadal WAT of *DKO* female mice at 12 weeks of age (n=2 mice) and (c) brain samples of healthy and Alzheimer's disease 5xFAD mice as negative and positive controls, respectively. Arrows, ThS-positive signals.

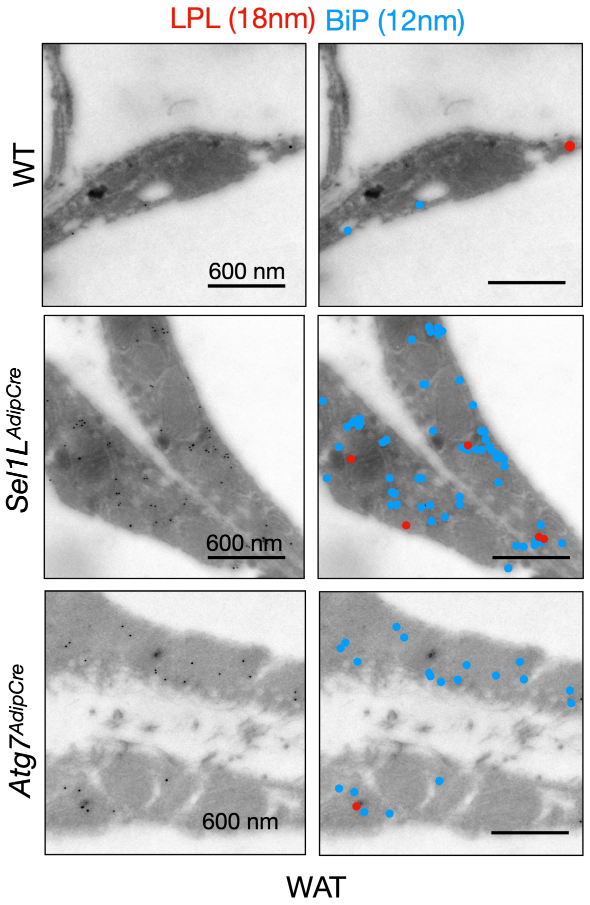

Supplementary Figure 9. LPL-BiP colocalization in WAT visualized using immunogold-TEM. Representative TEM images following immunogold co-labeling of LPL and BiP in gonadal WAT (n= 2-3 mice per genotype). Images with color coded gold particles against LPL (red) and BiP (blue) are shown on the right.

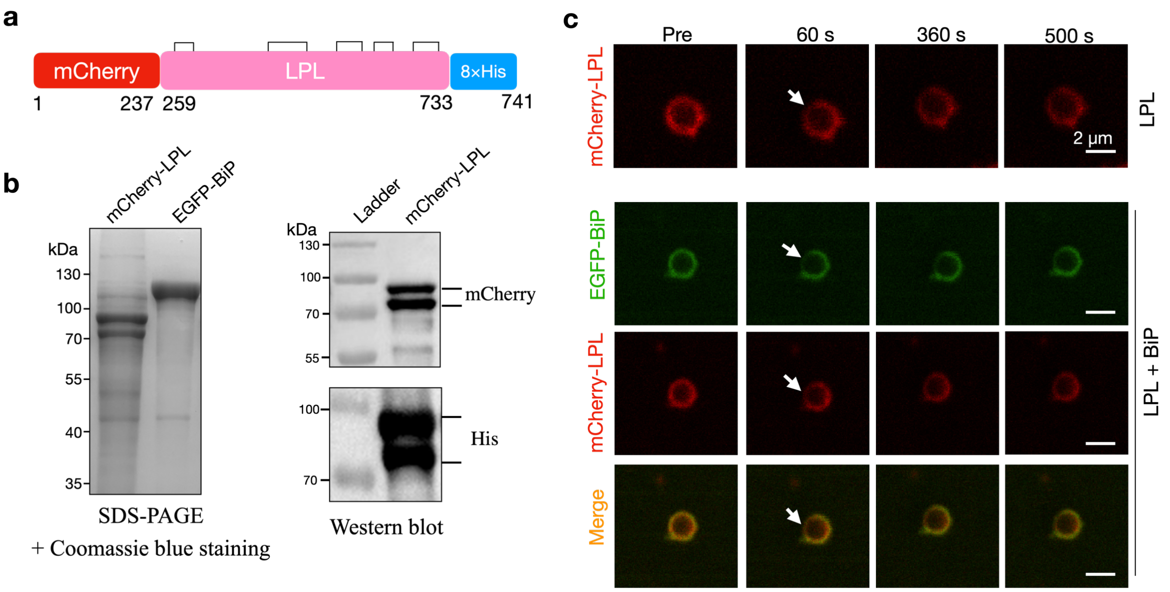

Supplementary Figure 10. ***In vitro* reconstitution of LPL condensates, coated by BiP.**
a Schematic diagram of mCherry-LPL-8xHis with the position of five intramolecular disulfide bonds shown on top. **b** Coomassie blue staining (left) and western blot analysis (right) of in vitro purified mCherry-LPL and/or EGFP-BiP (n=3 independent repeats). **c** Representative FRAP images of mCherry-LPL condensates *in vitro* in the presence or absence of EGFP-BiP (n=3 independent repeats). Arrows, bleached regions.

Supplementary Table 1. SEL1L IP-MS_ Protein hits enriched in $Hrd1^{Ucp1Cre}$ BAT.

		Peptide Spectrum Matches (PSMs)			
Protein	Protein name	IgG	$Sel1L^{Ucp1Cre}$	WT	$Hrd1^{Ucp1Cre}$
HSPA5	Endoplasmic reticulum chaperone BiP	3	40	0	226
HSP90B1	Endoplasmin	0	5	0	149
OS9	Protein OS-9	0	1	0	100
ERLEC1	Endoplasmic reticulum lectin 1	0	4	0	51
SERPINH1	Serpin H1	0	0	0	20
LPL	Lipoprotein lipase	0	1	0	13
PDIA6	Protein disulfide-isomerase A6	0	1	0	13
CES1D	Carboxylesterase 1D	1	0	0	8
ADIPOQ	Adiponectin	0	0	0	8
HSP90AB1	Heat shock protein HSP 90-beta	0	1	0	6
SMIM14	Small integral membrane protein 14	0	0	0	6
PGD	6-phosphogluconate dehydrogenase, decarboxylating	0	0	0	6
TKT	Transketolase	1	0	0	4
H2-D1	H-2 class I histocompatibility antigen, D-B alpha chain	0	0	0	4
SLC25A1	Tricarboxylate transport protein, mitochondrial	0	0	0	4
SDF2L1	Stromal cell-derived factor 2-like protein 1	0	0	0	4
GGCX	Vitamin K-dependent gamma-carboxylase	0	0	0	4
PHB	Prohibitin	0	0	0	4
EDEM1	ER degradation-enhancing alpha-mannosidase-like protein 1	0	0	0	4
SOD3	Extracellular superoxide dismutase [Cu-Zn]	0	0	0	4
H2-Q10	H-2 class I histocompatibility antigen, Q10 alpha chain	0	0	0	3
COLGALT1	Procollagen galactosyltransferase 1	0	0	0	3
RPN2	Dolichyl-diphosphooligosaccharide--protein glycosyltransferase subunit 2	0	0	0	3
H2-Q6	Class Ib MHC antigen Qa-2	0	0	0	3
H2-Q7	H-2 class I histocompatibility antigen, Q7 alpha chain	0	0	0	3

PLOD3	Multifunctional procollagen lysine hydroxylase and glycosyltransferase LH3	0	0	0	3
P4HA1	Prolyl 4-hydroxylase subunit alpha-1	0	0	0	3
POMT1	Protein O-mannosyl-transferase 1	0	0	0	3
PON3	Serum paraoxonase/lactonase 3	0	0	0	3
SEL1L	Protein sel-1 homolog 1	0	12	4	90
ATP5F1B	ATP synthase subunit beta, mitochondrial	0	0	0	2
SLC25A3	Phosphate carrier protein, mitochondrial	0	0	0	2
CTSD	Cathepsin D	0	0	0	2
DDOST	Dolichyl-diphosphooligosaccharide--protein glycosyltransferase 48 kDa subunit	0	0	0	2
STT3A	Dolichyl-diphosphooligosaccharide--protein glycosyltransferase subunit STT3A	0	0	0	2
HP	Haptoglobin	0	0	0	2
SLC25A11	Mitochondrial 2-oxoglutarate/malate carrier protein	0	0	0	2
GPX3	Glutathione peroxidase 3	0	0	0	2
ST3GAL6	Type 2 lactosamine alpha-2,3-sialyltransferase	0	0	0	2
AOC3	Membrane primary amine oxidase	0	0	0	2

The SEL1L IP-MS was performed using brown adipose tissue (BAT) from n=1 mouse per genotype.

Criteria:

- PSM in $Hrd1^{Ucp1Cre} \geq 2$
- PSM in $Hrd1^{Ucp1Cre}$ / PSM in WT ≥ 2
- PSM in $Hrd1^{Ucp1Cre}$ / PSM in $Sel1L^{Ucp1Cre} \geq 4$
- PSM in $Hrd1^{Ucp1Cre}$ / PSM in IgG ≥ 4
- Keratin proteins are excluded

Supplementary Table 2. LPL IP-MS_ Protein hits enriched in WT versus DKO WAT as the Cluster 1.

		Peptide Spectrum Matches (PSMs)						
		Experiment #1				Experiment #2		
Protein	Protein name	IgG	WT	Sel1L^{AdipCre}	DKO	IgG	WT	Atg7^{AdipCre}
Lpl	Lipoprotein lipase	0	48	57	98	0	231	218
Ogdh	2-oxoglutarate dehydrogenase, mitochondrial	0	18	5	9	0	86	119
Cav1	Caveolin-1	0	15	6	4	2	28	37
Acly	ATP-citrate synthase	0	11	1	2	0	20	26
Acaca	Acetyl-CoA carboxylase 1	0	10	2	1	1	61	83
Myh11	Myosin-11	0	9	2	1	0	45	17
Clu	Clusterin	0	7	1	0	0	23	19
Lrrfip1	Leucine-rich repeat flightless-interacting protein 1	0	7	4	1	0	70	57
Rpl18	60S ribosomal protein L18	0	6	0	0	4	24	21
Gphn	Gephyrin	0	5	0	0	0	6	17
Rpl7a	60S ribosomal protein L7a	0	5	2	1	0	32	35
Pdha1	Pyruvate dehydrogenase E1 component subunit alpha, somatic form, mitochondrial	0	5	3	2	0	6	12
Pzp	Pregnancy zone protein	0	4	2	0	0	15	5
Rpl3	60S ribosomal protein L3	0	4	0	0	0	47	37
Rpl6	60S ribosomal protein L6	0	4	0	0	0	28	33
Rpl4	60S ribosomal protein L4	0	4	0	0	0	57	59
Rpl29	60S ribosomal protein L29	0	4	3	1	0	8	8
H1-4	Histone H1.4	0	4	1	1	7	11	12
Cltc	Clathrin heavy chain 1	0	4	4	2	0	52	49
Rpl13	60S ribosomal protein L13	0	4	0	2	0	20	28
Rpl18a	60S ribosomal protein L18a	0	3	1	0	0	12	11

Hnrnpa 2b1	Heterogeneous nuclear ribonucleoproteins A2/B1	0	3	1	0	2	12	8
Rpl7	60S ribosomal protein L7	0	3	0	0	1	31	27
Rbm6	RNA-binding motif protein 6	0	3	0	0	0	19	26
Rps3	40S ribosomal protein S3	0	3	2	1	2	26	25
Plin4	Perilipin-4	0	3	1	1	0	33	29
Rps16	40S ribosomal protein S16	0	3	1	1	1	14	15
Cavin2	Caveolae-associated protein 2	0	3	1	1	2	23	20
Rpl34	60S ribosomal protein L34 (Fragment)	0	3	0	1	0	9	10
Rps6	40S ribosomal protein S6	0	3	0	1	1	31	30
Rpl10l	60S ribosomal protein L10-like	0	2	3	0	0	0	0
Mfap5	Microfibrillar-associated protein 5	0	2	2	0	0	2	2
Cyp2e1	Cytochrome P450 2E1	0	2	2	0	0	15	22
Myl6	Myosin light polypeptide 6	0	2	0	0	1	15	11
Itm2b	Integral membrane protein 2B	0	2	0	0	0	16	13
Vtn	Vitronectin	0	2	0	0	0	26	18
Rpl28	60S ribosomal protein L28	0	2	0	0	0	10	12
Nsf	Vesicle-fusing ATPase	0	2	0	0	0	5	12
Rps8	40S ribosomal protein S8	0	2	0	0	2	35	35
Rpl23a	60S ribosomal protein L23a	0	2	0	0	6	25	21
Rpl30	60S ribosomal protein L30	0	2	0	0	0	11	10
Esyt1	Extended synaptotagmin-1	0	2	0	0	0	16	22
Rpl21	60S ribosomal protein L21	0	2	0	0	0	5	5
Rpl37	60S ribosomal protein L37	0	2	0	0	0	5	5
Stom	Erythrocyte band 7 integral membrane protein	0	2	3	1	0	1	1
Igkv3-7	Immunoglobulin kappa variable 3-7	0	2	2	1	0	0	0
A2m	Alpha-2-macroglobulin-P	0	2	2	1	0	6	5

S100a10	Protein S100-A10	0	2	1	1	0	7	3

The LPL IP-MS was performed using white adipose tissue (WAT) pooled from n=5 WT, 6 Sel1L^{AdipCre}, and 7 DKO mice in Experiment #1; n=4 WT and 3 Atg7^{AdipCre} mice in Experiment #2.

Criteria:

- PSMs in WT ≥ 2
- PSMs in WT / PSMs in DKO ≥ 2
- PSMs in IgG =0
- Keratin proteins are excluded

Supplementary Table 3. LPL IP-MS_ Protein hits enriched in DKO versus WT WAT as the Cluster 2.

		Peptide Spectrum Matches (PSMs)						
		Experiment #1				Experiment #2		
Protein	Protein name	IgG	WT	Sel1L^{AdipCre}	DKO	IgG	WT	Atg7^{AdipCre}
Lpl	Lipoprotein lipase	0	48	57	98	0	231	218
Hspa5	Endoplasmic reticulum chaperone BiP	28	17	218	448	13	141	142
C4b	Complement C4-B	0	1	9	97	0	16	13
Hsp90b1	Endoplasmin	0	3	37	63	0	17	22
Os9	Protein OS-9	0	0	27	46	0	0	0
C3	Complement C3	0	5	3	42	0	22	15
Ugt1a6	UDP-glucuronosyltransferase 1-6	0	0	1	37	0	0	0
Ccpg1	Cell cycle progression protein 1	0	0	0	36	0	0	2
Gpx3	Glutathione peroxidase 3	0	0	3	35	0	2	0
Plod3	Multifunctional procollagen lysine hydroxylase and glycosyltransferase LH3	0	0	14	34	0	0	0
Rpn1	Dolichyl-diphosphooligosaccharide--protein glycosyltransferase subunit 1	0	1	2	34	0	6	8
Ugt1a6b	UDP-glucuronosyltransferase	0	0	1	33	0	0	0
Ugt1a7c	UDP-glucuronosyltransferase 1-7C	0	0	0	33	0	0	0
Mlec	Malectin	0	0	0	26	0	1	1
Adipoq	Adiponectin	0	0	8	23	0	4	4
Pgd	6-phosphogluconate dehydrogenase, decarboxylating	0	0	5	18	0	1	0
Prxl2a	Peroxiredoxin-like 2A	0	0	1	17	0	13	15
H6pd	GDH/6PGL endoplasmic bifunctional protein	0	0	0	16	0	0	2

Sts	Steryl-sulfatase	0	0	5	16	0	0	0
Ces1d	Carboxylesterase 1D	0	0	1	15	0	11	18
Dnajb9	DnaJ homolog subfamily B member 9	0	0	0	15	0	0	0
Dnajc3	DnaJ homolog subfamily C member 3	0	0	1	15	0	1	2
Entpd5	Ectonucleoside triphosphate diphosphohydrolase 5	0	0	1	15	0	0	2
H2-Q6	Class Ib MHC antigen Qa-2	0	3	7	15	0	0	0
P4ha1	Prolyl 4-hydroxylase subunit alpha-1	0	0	1	14	0	0	0
Rpn2	Dolichyl-diphosphooligosaccharide--protein glycosyltransferase subunit 2	0	0	0	14	0	5	4
Pdia4	Protein disulfide-isomerase A4	0	1	9	14	0	1	9
Atp2a2	Sarcoplasmic/endoplasmic reticulum calcium ATPase 2	0	2	0	14	0	12	30
Aoc3	Membrane primary amine oxidase	0	3	8	14	0	37	20
Ddost	Dolichyl-diphosphooligosaccharide--protein glycosyltransferase 48 kDa subunit	0	0	1	13	0	3	4
Man2a2	Alpha-mannosidase 2x	0	0	1	13	0	0	0
Npr3	Atrial natriuretic peptide receptor 3	0	0	0	13	0	5	2
P4ha1	Prolyl 4-hydroxylase subunit alpha-1	0	0	0	13	0	0	0
Ces1f	Carboxylesterase 1F	0	0	0	12	0	1	2
Magt1	Magnesium transporter protein 1	0	0	1	12	0	1	1
Mmp28	Matrix metallopeptidase 28 (Epilysin)	0	0	3	12	0	0	0
Naga	Alpha-N-acetylgalactosaminidase	0	0	0	12	0	0	0
Uggt1	UDP-glucose:glycoprotein glucosyltransferase 1	0	0	1	12	0	4	13

H2-Q4	Histocompatibility 2, Q region locus 4	0	3	5	12	0	0	0
Calr	Calreticulin	0	0	0	11	0	2	3
Pdia3	Protein disulfide-isomerase A3	0	0	6	11	0	8	14
Plod2	Procollagen-lysine,2-oxoglutarate 5-dioxygenase 2	0	0	6	11	0	0	0
Pon3	Serum paraoxonase/lactonase 3	0	0	0	11	0	0	0
Sdf2l1	Stromal cell-derived factor 2-like protein 1	0	0	4	11	0	0	0
P4hb	Protein disulfide-isomerase	0	0	3	10	0	6	6
Tsku	Tsukushin	0	0	0	10	0	0	0
S100a11	Protein S100-A11	0	2	5	10	2	8	12
Colgalt1	Procollagen galactosyltransferase 1	0	0	0	9	0	0	0
Ssr4	Signal sequence receptor, delta	0	0	0	9	0	0	0
Stt3a	Dolichyl-diphosphooligosaccharide--protein glycosyltransferase subunit STT3A	0	0	1	9	0	0	1
Chpf2	Hexosyltransferase	0	0	0	8	0	0	0
Erlec1	Endoplasmic reticulum lectin 1	0	0	5	8	0	0	0
Hsd11b1	Corticosteroid 11-beta-dehydrogenase isozyme 1	0	0	0	8	0	0	3
Man2a1	Alpha-mannosidase 2	0	0	0	8	0	0	0
P4ha2	Prolyl 4-hydroxylase subunit alpha-2	0	0	1	8	0	0	0
Sdf2	Stromal cell-derived factor 2	0	0	1	8	0	0	1
Sod3	Extracellular superoxide dismutase [Cu-Zn]	0	0	1	8	0	0	0
Lrp1	Prolow-density lipoprotein receptor-related protein 1	0	2	5	8	0	97	77
Emc1	ER membrane protein complex subunit 1	0	0	1	7	0	2	2

Ext2	Exostosin-2	0	0	1	7	0	0	0
Ggcx	Vitamin K-dependent gamma-carboxylase	0	0	0	7	0	0	0
Ltbp3	Latent-transforming growth factor beta-binding protein 3	0	0	5	7	0	0	0
Stt3b	Dolichyl-diphosphooligosaccharide--protein glycosyltransferase subunit STT3B	0	0	2	7	0	0	3
Txndc12	Thioredoxin domain-containing protein 12	0	0	0	7	0	0	0
Chrdl1	Chordin-like protein 1	0	0	0	6	0	0	0
Ctsd	Cathepsin D	0	0	2	6	0	0	0
Dnajb11	DnaJ homolog subfamily B member 11	0	0	1	6	0	0	2
Edem3	ER degradation-enhancing alpha-mannosidase-like protein 3	0	0	0	6	0	0	0
Ganab	Neutral alpha-glucosidase AB	0	0	0	6	0	1	2
Olfml1	Olfactomedin-like 1	0	0	3	6	0	0	0
Tmed2	Transmembrane emp24 domain-containing protein 2 (Fragment)	0	0	0	6	0	0	0
Cp	Ceruloplasmin	0	0	1	5	0	4	1
Dnajc10	DnaJ homolog subfamily C member 10	0	0	1	5	0	0	0
Mettl7a1	Methyltransferase-like 7A1	0	0	0	5	0	6	10
Naxe	NAD(P)H-hydrate epimerase	0	0	0	5	0	0	0
Ogfod3	2-oxoglutarate and iron-dependent oxygenase domain-containing protein 3	0	0	0	5	0	0	0
Spcs3	Signal peptidase complex subunit 3	0	0	0	5	0	0	0
Ttc17	Tetratricopeptide repeat protein 17	0	0	0	5	0	0	0
Vapa	Vesicle-associated membrane protein-associated protein A	0	1	0	5	0	6	7

Adpgk	ADP-dependent glucokinase	0	0	0	4	0	0	0
Asph	Aspartyl/asparaginyl beta-hydroxylase	0	0	1	4	0	5	10
Atad3	ATPase family AAA domain-containing protein 3	0	0	0	4	0	15	29
Bgn	Biglycan	0	0	0	4	0	12	10
Cyb5r1	NADH-cytochrome b5 reductase 1 (Fragment)	0	0	0	4	0	0	0
Edem1	ER degradation-enhancing alpha-mannosidase-like protein 1	0	0	0	4	0	0	0
Ermp1	Endoplasmic reticulum metallopeptidase 1	0	0	0	4	0	0	0
Ero1a	ERO1-like protein alpha	0	0	0	4	0	0	0
Fam3a	Protein FAM3A	0	0	0	4	0	0	0
Galnt2	Polypeptide N-acetylgalactosaminyltransferase 2	0	0	0	4	0	0	0
Glb1l2	Beta-galactosidase	0	0	0	4	0	0	7
Hsd17b12	Very-long-chain 3-oxoacyl-CoA reductase	0	0	0	4	5	9	26
Igf2r	Cation-independent mannose-6-phosphate receptor	0	0	0	4	0	0	0
Itgav	Integrin alpha-V	0	0	0	4	0	0	0
Ndufa4	Cytochrome c oxidase subunit NDUFA4	0	0	2	4	0	4	13
Nomo1	Nodal modulator 1	0	0	0	4	0	0	0
Nrbp2	Nuclear receptor-binding protein 2	0	0	0	4	0	0	0
Plod1	Procollagen-lysine,2-oxoglutarate 5-dioxygenase 1	0	0	1	4	0	1	0
Sec61a1	Protein transport protein Sec61 subunit alpha isoform 1	0	0	1	4	0	1	1
Spcs2	Signal peptidase complex subunit 2	0	0	0	4	0	0	0
Tmem43	Transmembrane protein 43	0	0	0	4	0	3	10

Vcp	Transitional endoplasmic reticulum ATPase	0	0	4	4	0	7	6
Canx	Calnexin	0	1	3	4	0	12	13
Sqor	Sulfide:quinone oxidoreductase, mitochondrial	0	1	1	4	0	18	26

The LPL IP-MS was performed using white adipose tissue (WAT) pooled from n=5 WT, 6 Sel1L^{AdipCre}, and 7 DKO mice in Experiment #1; n=4 WT and 3 Atg7^{AdipCre} mice in Experiment #2.

Criteria:

- PSMs in DKO ≥ 4
- PSMs in DKO / PSMs in WT ≥ 4
- PSMs in IgG = 0
- Keratin proteins are excluded
- HSPA5 was added back due to its high abundance in *DKO* group, large fold change of PSM in *DKO* versus PSM in WT and its known function in protein folding in the ER.
