# Supplementary material for: The mechanisms to dispose of misfolded proteins in the endoplasmic reticulum of adipocytes
Source: Nat Commun. 2023 May 30;14:3132. doi: 10.1038/s41467-023-38690-4 (PMC10229581; doi:10.1038/s41467-023-38690-4)
Supplement: Supplementary file 3 — Description of Additional Supplementary Files [file 41467_2023_38690_MOESM3_ESM.pdf]

Description of Additional Supplementary File

Supplementary Video 1. 3D FIB-SEM image series showing reconstruction of CERFs in DKO adipocytes. Serial SEM images (370 sections, 10 nm/interval) of gonadal WAT were obtained from DKO mice. One of the SEM images was also shown in Fig. 2g. CERF is the round structure with high electron density in the middle of the section. The video were made by using Imaris software x64 9.5.1
