# Supplementary material for: The mechanisms to dispose of misfolded proteins in the endoplasmic reticulum of adipocytes
Source: Nat Commun. 2023 May 30;14:3132. doi: 10.1038/s41467-023-38690-4 (PMC10229581; doi:10.1038/s41467-023-38690-4)
Supplement: Supplementary file 5 — Reporting Summary [file 41467_2023_38690_MOESM5_ESM.pdf]

nature portfolio

Corresponding author(s): Ling Qi, Shuangcheng Wu

Last updated by author(s): Apr 26, 2023

Reporting Summary

Nature Portfolio wishes to improve the reproducibility of the work that we publish. This form provides structure for consistency and transparency in reporting. For further information on Nature Portfolio policies, see our Editorial Policies and the Editorial Policy Checklist.

Statistics

For all statistical analyses, confirm that the following items are present in the figure legend, table legend, main text, or Methods section.

n/a	Confirmed	
☐	☒	The exact sample size (*n*) for each experimental group/condition, given as a discrete number and unit of measurement
☐	☒	A statement on whether measurements were taken from distinct samples or whether the same sample was measured repeatedly
☐	☒	The statistical test(s) used AND whether they are one- or two-sided<br>*Only common tests should be described solely by name; describe more complex techniques in the Methods section.*
☒	☐	A description of all covariates tested
☐	☒	A description of any assumptions or corrections, such as tests of normality and adjustment for multiple comparisons
☐	☒	A full description of the statistical parameters including central tendency (e.g. means) or other basic estimates (e.g. regression coefficient) AND variation (e.g. standard deviation) or associated estimates of uncertainty (e.g. confidence intervals)
☐	☒	For null hypothesis testing, the test statistic (e.g. *F*, *t*, *r*) with confidence intervals, effect sizes, degrees of freedom and *P* value noted<br>*Give P values as exact values whenever suitable.*
☒	☐	For Bayesian analysis, information on the choice of priors and Markov chain Monte Carlo settings
☒	☐	For hierarchical and complex designs, identification of the appropriate level for tests and full reporting of outcomes
☒	☐	Estimates of effect sizes (e.g. Cohen's *d*, Pearson's *r*), indicating how they were calculated

Our web collection on statistics for biologists contains articles on many of the points above.

Software and code

Policy information about availability of computer code

Data collection	Confocal data were collected using Nikon A1 and Leica STELLARIS 8 FALCON Confocal Microscope; H&E images of data were collected using Aperio Imagescope software v102.0.4.6; Western Blot data were collected by Image Lab software 4.1 (Bio-rad); the EM data of adipose tissue were collected using JEOL JEM-1400 Plus LaB6 TEM; and the negative staining of LPL-BiP droplets was collected using Hitachi HT7800 transmission electron microscope; FIB-SEM data were collected using FEI Helios Nanolab 650 DualBeam. The details were described in the Method section of the manuscript.
Data analysis	Following software were used in this study: Image Lab software 4.1 (Bio-rad), ImageJ2 version 2.9.0, Avizo v.9.3 (Thermo Fisher Scientific), Imaris x64 9.5.1 (Oxford Instruments), Aivia 10.5.1 (Leica Microsystems), and Proteome Discoverer (v2.4, Thermo Scientific). Statistics analysis was performed by Graphpad Prism8. The details were described in the Method section of the manuscript.

For manuscripts utilizing custom algorithms or software that are central to the research but not yet described in published literature, software must be made available to editors and reviewers. We strongly encourage code deposition in a community repository (e.g. GitHub). See the Nature Portfolio guidelines for submitting code & software for further information.

Data

Policy information about availability of data

All manuscripts must include a data availability statement. This statement should provide the following information, where applicable:

- Accession codes, unique identifiers, or web links for publicly available datasets
- A description of any restrictions on data availability
- For clinical datasets or third party data, please ensure that the statement adheres to our policy

Proteomics datasets have been deposited to the ProteomeXchange Consortium via the PRIDE partner repository with the dataset identifier PXD038310 and PXD040899. The predicted structure of LPL is available as open data via PDB ID: 6U7B. Other data supporting the findings of this study are available within the article and the supplementary information. Source data are provided with the paper.

Human research participants

Policy information about studies involving human research participants and Sex and Gender in Research.

Reporting on sex and gender	N/A
Population characteristics	N/A
Recruitment	N/A
Ethics oversight	N/A

Note that full information on the approval of the study protocol must also be provided in the manuscript.

Field-specific reporting

Please select the one below that is the best fit for your research. If you are not sure, read the appropriate sections before making your selection.

☒ Life sciences ☐ Behavioural & social sciences ☐ Ecological, evolutionary & environmental sciences

For a reference copy of the document with all sections, see nature.com/documents/nr-reporting-summary-flat.pdf

Life sciences study design

All studies must disclose on these points even when the disclosure is negative.

Sample size	Sample size was determined based on previous similar experiments (Sha et al., Cell metabolism 2014; Zhou et al., Science 2020 and Shrestha et al., JCI 2023), and by the formula of the power analysis, N=8(CV)^2[1+(1-PC)^2]/(PC)^2, to reach the error = 0.05, Power = 0.80, percentage change in means (PC) = 20%, co-efficient of variation (CV) = 10 ~ 15% (varies between the experiments). The exact number of biologically independent mice and independent experiments for in vitro studies were indicated in the figure legend.
Data exclusions	No animals or samples were excluded from the analysis.
Replication	Most experiments (except IP-MS) have been repeated at least twice with multiple independent samples. All attempts for replication were successful. The experiment of SEL1L IP-MS were performed once while LPL IP-MS was performed once with pooled tissues from more than three mice. The details were indicated in the figure legend, Methods section and supplementary tables. Source data was provided with the paper.
Randomization	Mice were randomly assigned based on the age, genotype and gender. Cells were grown under the same conditions and randomly allocated into different groups without any bias.
Blinding	Mice were genotypes and ear tagged with serial number which were used to mark the serum and tissue samples. Investigators were blinded during group allocation and data collection. The genotypes of sample were only identified once the final data were analyzed. The number of autophagic vacuoles (AVs) were counted by a blinded investigator.

Reporting for specific materials, systems and methods

We require information from authors about some types of materials, experimental systems and methods used in many studies. Here, indicate whether each material, system or method listed is relevant to your study. If you are not sure if a list item applies to your research, read the appropriate section before selecting a response.

Materials & experimental systems

n/a	Involved in the study
☐	☒ Antibodies
☐	☒ Eukaryotic cell lines
☒	☐ Palaeontology and archaeology
☐	☒ Animals and other organisms
☒	☐ Clinical data
☒	☐ Dual use research of concern

Methods

n/a	Involved in the study
☒	☐ ChIP-seq
☒	☐ Flow cytometry
☒	☐ MRI-based neuroimaging

Antibodies

Antibodies used

Western blot antibodies: anti-HSP90 (1:10,000, Santa Cruz, sc-7947), anti-GAPDH (1:10,000, ProteinTech, 60004-1-Ig), anti-LPL (1:500, gift from Dr. Andre Bensadoun, Cornell University and R&D Systems AF7197-SP), anti-SEL1L (1:1000, Abcam, ab78298 and 1:10,000, homemade), anti-HRD1 (1:200, gift from Richard Wojcikiewicz, Syracuse University and 1:1000, ProteinTech, 13473-1-AP), anti-ATG7 (1:1000, Cell Signaling, 8558S), anti-P62 (1:5000, MBL, PM066 and 1:5000, Enzo, BML-PW9860), anti-LC3B (1:2000, Cell Signaling, 2775S), anti-BiP (1:2000, Abcam ab21685), anti-OS9 (1:2000, Abcam, ab109510), anti-GLUT4 (1:2000, ProteinTech, 66846-I-Ig), anti-CALNEXIN (1:5000, ProteinTech, 10427-2-AP), anti-MLEC (1:2000, ProteinTech, 26655-1-AP), anti-PLOD3 (1:2000, ProteinTech,11027-1-AP), anti-CAV1 (1:2000, ABclonal, A1555), anti-CASPASE 3 (1:1000, Cell Signaling, 9662S); anti-Cleaved CASPASE 3 (1:1000, Cell Signaling, 9661S), anti-H2A (1:1000, Cell Signaling, 2578), anti-mCherry tag (1:1000, ABclonal, AE002), and anti-His tag (1:1000, ABclonal, AE003). Secondary antibodies: donkey anti-goat IgG-HRP (1:5000, ThermoFisher, #PA1-28664), goat anti-rabbit IgG-HRP (1:5000, Bio-Rad, #1706515), goat anti-mouse IgG-HRP (1:5000, Bio-Rad, #1706516) and goat anti-guinea pig IgG-HRP (1:5000, Bio-Rad, #AHP863P). Secondary antibodies used in immunoprecipitation western blot: Trueblot anti-goat IgG HRP (1:1000, Rockland, mouse monoclonal eB270, #18-8814-31, for detecting LPL in LPL IP). For 1mg protein, 4µg IgG (MBL, PM094 or Cell Signaling, #2729) were used.

Antibodies for immunofluorescent staining: anti-LPL (1:200, gift from Dr. Andre Bensadoun, Cornell University, and 1:100, R&D Systems AF7197-SP), anti-GPIHBP1 (1:250, gift from Dr. Stephen G Young, University of California, Los Angeles); anti-KDEL (1:500, Novus NBP1-97469), anti-BiP (1:500, Abcam ab21685), anti-P62 (1:500, MBL, PM066), anti-P62 (1:500, Enzo, BML-PW9860), anti-OS9 (1:250, Abcam, ab109510), anti-PLOD3 antibody (1:500, ProteinTech,11027-1-AP), anti-CAV1 (1:250, ABclonal, A1555), anti-MLEC (1:200, ProteinTech, 26655-1-AP). Secondary antibodies: Alexa fluor 488 affinipure donkey anti-goat IgG (H+L) (1:500 Jackson ImmunoResearch, 705-546-147); Alexa fluor plus 555 donkey anti-goat IgG (H+L) (1:500, Invitrogen, A32816); Alexa fluor plus 647 donkey anti-goat IgG (H+L) (1:500, Invitrogen, A32849); Alexa fluor 647 affinipure donkey anti-rat IgG (H+L) (1:500, Jackson ImmunoResearch, 712-606-150); Alexa fluor plus 555 donkey anti-mouse IgG (H+L) (1:500, Invitrogen, A32773); Alexa fluor 488 affinipure donkey anti-rabbit IgG (H+L) (1:500, Jackson ImmunoResearch, 711-546-152); Alexa fluor plus 555 donkey anti-rabbit IgG (H+L) (1:500, Invitrogen, A32794); Alexa fluor 647 affinipure donkey anti-rabbit IgG (H+L) (1:500, Jackson ImmunoResearch, 711-606-152); and Alexa fluor 647 affinipure donkey anti-guinea pig IgG (H+L) (1:500, Jackson ImmunoResearch, 706-606-148).

Antibodies for immunogold staining: anti-LPL (1:70, gift from Dr. Andre Bensadoun, Cornell University), anti-LPL (1:50, R&D Systems AF7197-SP), anti-KDEL (1:50, Novus NBP1-97469), anti-BiP (1:50, Abcam ab21685) and anti-P62 (1:50, Enzo, BML-PW9860). Secondary antibodies: 18 nm Colloidal Gold-conjugated AffiniPure Donkey Anti-Rabbit IgG (H+L) (1:25 , Jackson ImmunoResearch, #711-215-152); 12 nm Colloidal Gold AffiniPure Donkey Anti-Rabbit IgG (H+L) (1:25 , Jackson ImmunoResearch, #711-205-152); 12 nm Colloidal Gold AffiniPure Donkey Anti-Mouse IgG (H+L) (1:25 , Jackson ImmunoResearch, #715-205-150); 18 nm Colloidal Gold AffiniPure Donkey Anti-Goat IgG (H+L) (1:25 , Jackson ImmunoResearch, #705-215-147).

Validation

anti-LPL (homemade) has been validated for Western blot and immunostaining in mouse.
References:
Sha, Haibo, Shengyi Sun, Adam B. Francisco, Nicole Ehrhardt, Zhen Xue, Lei Liu, Peter Lawrence et al. "The ER-associated degradation adaptor protein Sel1L regulates LPL secretion and lipid metabolism." Cell metabolism 20, no. 3 (2014): 458-470.
Davies, Brandon SJ, Anne P. Beigneux, Richard H. Barnes, Yiping Tu, Peter Gin, Michael M. Weinstein, Chika Nobumori et al. "GPIHBP1 is responsible for the entry of lipoprotein lipase into capillaries." Cell metabolism 12, no. 1 (2010): 42-52.

anti-SEL1L (homemade) has been validated for Western blot (Zhou et al., Science 2020) in mouse.
Reference:
Zhou, Zhangsen, Mauricio Torres, Haibo Sha, Christopher J. Halbrook, Françoise Van den Bergh, Rachel B. Reinert, Tatsuya Yamada et al. "Endoplasmic reticulum–associated degradation regulates mitochondrial dynamics in brown adipocytes." Science 368, no. 6486 (2020): 54-60.

anti-GPIHBP1 (Homemade) has been validated for immunostaining in mouse.
Reference:
Davies, Brandon SJ, Anne P. Beigneux, Richard H. Barnes, Yiping Tu, Peter Gin, Michael M. Weinstein, Chika Nobumori et al. "GPIHBP1 is responsible for the entry of lipoprotein lipase into capillaries." Cell metabolism 12, no. 1 (2010): 42-52.

anti-HRD1 (gift from Richard Wojcikiewicz, Syracuse University) has been validated for Western blot in mouse.
Reference:
Sha, Haibo, Shengyi Sun, Adam B. Francisco, Nicole Ehrhardt, Zhen Xue, Lei Liu, Peter Lawrence et al. "The ER-associated degradation adaptor protein Sel1L regulates LPL secretion and lipid metabolism." Cell metabolism 20, no. 3 (2014): 458-470.

Remaining commercial antibodies have been validated by the manufactures:
anti-SEL1L antibody (Abcam, ab78298): validated for western blot by the manufacture (https://www.abcam.com/sel1l-antibody-ab78298.html) and previously (Sha, Haibo, Shengyi Sun, Adam B. Francisco, Nicole Ehrhardt, Zhen Xue, Lei Liu, Peter Lawrence et al.

"The ER-associated degradation adaptor protein Sel1L regulates LPL secretion and lipid metabolism." Cell metabolism 20, no. 3 (2014): 458-470.)

anti- LPL (R&D Systems AF7197): validated for western blot and immunostaining in mouse and human by the manufacture (https://www.rndsystems.com/products/human-mouse-lipoprotein-lipase-lpl-antibody_af7197).

anti-HRD1 (ProteinTech, 13473-1): validated for western blot, immunostaining and immunoprecipitation in mouse and human by the manufacture (https://www.ptglab.com/products/SYVN1-Antibody-13473-1-AP.htm).

anti-ATG7 Cell Signaling, 8558S): validated for western blot in mouse and human by the manufacture (https://www.cellsignal.com/products/primary-antibodies/atg7-d12b11-rabbit-mab/8558).

anti-P62 (MBL, PM066): validated for western blot, immunostaining and immunoprecipitation in mouse by the manufacture (https://www.mblbio.com/bio/g/dtl/A/?pcd=PM066).

anti-P62 (Enzo, BML-PW9860): validated for western blot and immunostaining by the manufacture (https://www.enzolifesciences.com/BML-PW9860/p62-human-polyclonal-antibody/).

anti-LC3B(Cell Signaling, 2775S): validated for western blot in mouse and human by the manufacture (https://www.cellsignal.com/products/primary-antibodies/lc3b-antibody/2775).

anti-BiP (Abcam ab21685): validated for western blot and immunostaining in mouse and human by the manufacture (https://www.abcam.com/grp78-bip-antibody-ab21685.html).

anti-KDEL (Novus NBP1-97469): validated for immunostaining in mouse and human by the manufacture (https://www.novusbio.com/products/kdel-antibody-10c3_nbp1-97469).

anti-OS9 (Abcam, ab109510): validated for western blot and immunostaining in mouse and human by the manufacture (https://www.abcam.com/os9-antibody-epr42722-ab109510.html).

anti-GLUT4 (ProteinTech, 66846-I-Ig): validated for western blot in mouse and human by the manufacture (https://www.ptglab.com/products/GLUT4-Antibody-66846-1-Ig.htm).

anti-CALNEXIN (ProteinTech, 10427-2-AP): validated for western blot in mouse and human by the manufacture (https://www.ptglab.com/products/CANX-Antibody-10427-2-AP.htm).

anti-MLEC: validated for western blot in mouse and human by the manufacture (https://www.ptglab.com/products/Malectin-Antibody-26655-1-AP.htm), we further validated its immunostaining application in mouse in this study.

anti-PLOD3 (ProteinTech,11027-1-AP): validated for western blot and immunostaining in mouse and human by the manufacture (https://www.ptglab.com/products/PLOD3-Antibody-11027-1-AP.htm).

anti-CAV1 (ABclonal, A1555): validated for western blot and immunostaining in mouse and human by the manufacture (https://abclonal.com/catalog-antibodies/CAV1RabbitpAb/A1555).

anti-CASPASE 3 (Cell Signaling, 9662S): validated for western blot in mouse and human by the manufacture (https://www.cellsignal.com/products/primary-antibodies/caspase-3-antibody/9662).

anti-Cleaved CASPASE 3 (Cell Signaling, 9661S): validated for western blot in mouse and human by the manufacture (https://www.cellsignal.com/products/primary-antibodies/cleaved-caspase-3-asp175-antibody/9661).

anti-H2A (Cell Signaling, 2578): validated for western blot in mouse and human by the manufacture (https://www.cellsignal.com/products/primary-antibodies/histone-h2a-antibody-ii/2578).

anti-mCherry tag (ABclonal, AE002): validated for western blot by the manufacture (https://abclonal.com/catalog-antibodies/MouseantimCherryTagmAb/AE002).

anti-His tag (ABclonal, AE003): validated for western blot by the manufacture (https://abclonal.com/catalog-antibodies/MouseantiHisTagmAb/AE003).

anti-HSP90 (Santa Cruz, sc-7947): validated for western blot in human and mouse by the manufacture (https://www.scbt.com/p/hsp-90alpha-beta-antibody-h-114)

anti-GAPDH (ProteinTech, 60004-1-Ig): validated for western blot in human and mouse by the manufacture (https://www.ptglab.com/products/GAPDH-Antibody-60004-1-Ig.htm).

Eukaryotic cell lines

Policy information about cell lines and Sex and Gender in Research

Cell line source(s)	Pre-adipocytes were isolated from mouse brown adipose tissue and immortalized using SV40 large T antigen and differentiated into adipocytes, which was indicated in the Method section of the manuscript and reported previously (Zhou et al. Science 2022, and Klein et al., Bioessays 2002).<br>HEK293T were purchased from ATCC.

Authentication	The differentiated adipocytes were authenticated using visual examination of cell morphology, and accumulation of lipid droplets. The morphology of HEK293T cells were visually authenticated with microscope before use.
Mycoplasma contamination	All cell lines used for experiments had no mycoplasma contamination after testing.
Commonly misidentified lines (See ICLAC register)	The cell lines are not listed in the database.

Animals and other research organisms

Policy information about studies involving animals; ARRIVE guidelines recommended for reporting animal research, and Sex and Gender in Research

Laboratory animals	Species: Mus musculus; Sex: Female/Male; Age: 10-12weeks; 5 and 12 weeks in age-related studies (Fig.1h, 5c and 5e). Information of sex and age was indicated in the figure legends.<br>Strains:<br>1. All mice were in C57BL/6J background (Sha et al., Cell Metabolism 2014).<br>2. Sel1L flox/flox, Adip-Cre mice: Zhou et al., Science 2020 and Sha et al., Cell Metabolism 2014.<br>3. Atg7 flox/flox mice: Shrestha et al., JCI 2023, Shrestha et al., JCI 2020 and Eguchi et al., Cell Metabolism 2011. The mice were crossed with adiponectin promoter-driven Cre mice to generate Atg7 flox/flox, Adip-Cre mice.<br>4. Sel1L flox/flox, Atg7 flox/flox: Shrestha et al., JCI 2023. The mice were crossed with adiponectin promoter-driven Cre mice to generate Sel1L flox/flox;Atg7 flox/flox; Adip-Cre mice.<br>5. Sel1LERCre mice: Zhou et al., Science 2020 and Sun et al., PNAS 2014.<br>The details were also described in the Method section of the manuscript.
Wild animals	This study did not include wild animals.
Reporting on sex	Both males and females were used.
Field-collected samples	This study did not include field-collected samples.
Ethics oversight	All animal procedures were approved by and done in accordance with the IACUC at the University of Michigan Medical School (PRO00010658).

Note that full information on the approval of the study protocol must also be provided in the manuscript.
